# Supplementary material for: Evaluating spatiotemporal dynamics of snakebite in Sri Lanka: Monthly incidence mapping from a national representative survey sample
Source: PLoS Negl Trop Dis. 2021 Jun 1;15(6):e0009447. doi: 10.1371/journal.pntd.0009447 (PMC8195360; doi:10.1371/journal.pntd.0009447)
Supplement: S1 Appendix — (DOCX) [file pntd.0009447.s001.docx]

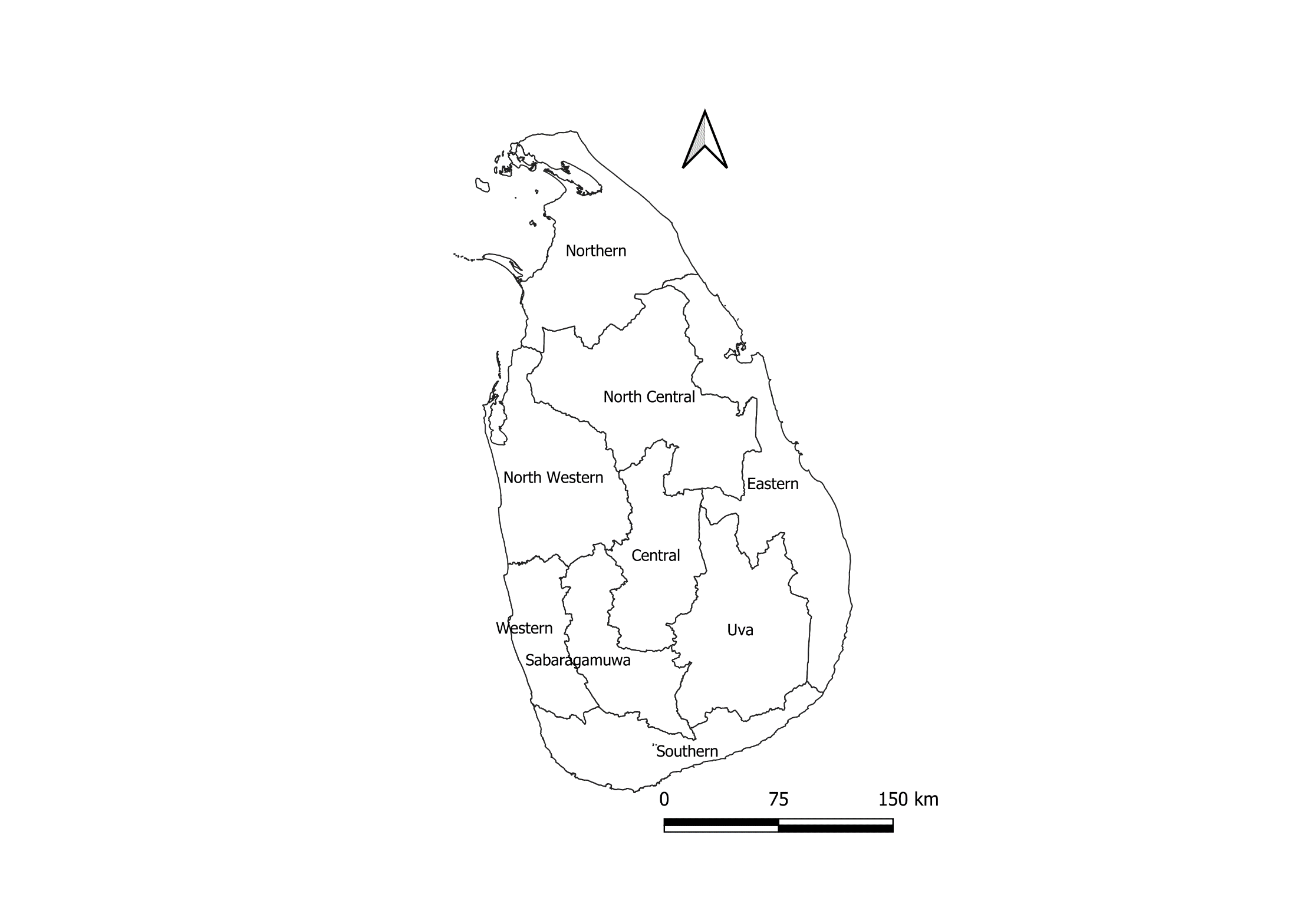


**S1 Appendix: Provinces of Sri Lanka.** Base layer of the map was obtained from the Survey Department of Sri Lanka (https://www.survey.gov.lk/).
