# Supplementary material for: Evaluating spatiotemporal dynamics of snakebite in Sri Lanka: Monthly incidence mapping from a national representative survey sample
Source: PLoS Negl Trop Dis. 2021 Jun 1;15(6):e0009447. doi: 10.1371/journal.pntd.0009447 (PMC8195360; doi:10.1371/journal.pntd.0009447)
Supplement: S2 Appendix — (PDF) [file pntd.0009447.s002.pdf]

Technical appendix

Spatiotemporal modelling of snakebite

Here, we give details of our formulation and fitting of statistical models to our Sri Lanka-wide spatio-temporal data. All computations used `R`, version 3.5.1 (`www.r-project.org`).

Definitions

- Snakebite is a bite event from any snake, irrespective of envenoming status.
- Envenoming bite is a snakebite with significant envenoming, which is defined as the presence of local tissue necrosis at the site of the bite, presence of neurotoxicity or bleeding manifestations
- Bite refers to both snakebite and envenoming bite to avoid repetition in the methods description, as the same statistical methods were applied to model both snakebite and envenoming bites.
- Survey month is the month in which the survey was conducted. The survey was conducted for 11 consecutive survey months, August 2012 to June 2013.
- Bitten month is the month in which the victim actually experienced a snakebite. The survey reported bites belong to 23 bitten months, August 2011 to June 2013.
- Recall time is the time difference between the bitten month and survey month of a bite. It ranged from 0 to 12 months.

Survey design

Sri Lanka is divided into 14,022 Grama Niladhari (GN) divisions, which were considered as *clusters* for data collection. In each province, 125 *clusters* were proportionally allocated among districts based on the size of the population within each district. In each *cluster*, 40 consecutive households were selected for the survey, with the initial household randomly selected using the electoral register as the sampling frame. Data were collected using an interviewer-administered two-part questionnaire. The first part obtained socio-demographic data from the households whilst the second part obtained details of snakebite events that occurred within the preceding 12 months. The questionnaire was pre-tested in each province, fine-tuned prior to use and translated into both official languages of Sri Lanka; Sinhala and Tamil. Trained data collectors were used to collect data. Data collection was facilitated by local volunteers. Face-to-face interviews were held with an adult member of the household. The survey was conducted from August 2012 to June 2013.

Our data-set has been compiled from 11 repeated cross-sectional surveys. In each survey, respondents were asked whether they had experienced a bite in the month of survey or within the 12 months preceding the survey month, and if so in which of these 13 months the bite occurred. The data therefore give reported bite frequencies over 23 consecutive months, at a time-resolution of one month.

Statistical model

Multivariate Poisson process

Our goal is to describe the rate at which snake-bite events occur over time in each of the 1,118 clusters. Our working assumption is that in each cluster, bites occur independently of each other. A statistical model for a process of this kind is called a *multivariate Poisson process.*

A multivariate Poisson point process of dimension r is completely specified by a set of r *intensity functions*, $\lambda_1(t), \lambda_2(t), ..., \lambda_r(t)$, where t denotes time. In our case, time runs over a period of 23 months and $r = 1,118$, the number of clusters. Each intensity function determines the rate at which events (here, reported snake bites or envenoming bites) occur in the corresponding cluster. In a Poisson process with intensity $\lambda(t)$, the number of events occurring between times a and b follows a Poisson distribution with expectation $\int_a^b \lambda(t)dt$.

For reasons to be explained, it is convenient to index each cluster by the province in which it is situated, hence we write the complete set of intensity functions as

$$\lambda_{pc}(t) : c = 1, ..., m_p; p = 1, ..., 9 \tag{1}$$

where p denotes province, c denotes cluster within province and m_p is the number of clusters within province p. Note also that each $\lambda_{pc}(t)$ can be decomposed into a sum of intensity functions,

$$\lambda_{pc}(t) = \sum_{s=13}^{g(t)} \lambda_{pc}(s, t) \tag{2}$$

where $g(t) =$ t +12 and $\lambda_{pc}(s, t)$ denotes the intensity for bites *reported* to have occurred in in cluster pc in month $t = 1, 2, ..., 23$ that are recorded in survey month $s = 13, 14, ..., 23$. Each cluster was sampled only once in the NSS but provided information on risk of bites for 13 of the 23 months; see Figure S1.

Figure S1. Survey design. The x-axis shows the range of months over which bites were reported. The y-axis shows the month in which the corresponding survey was conducted.

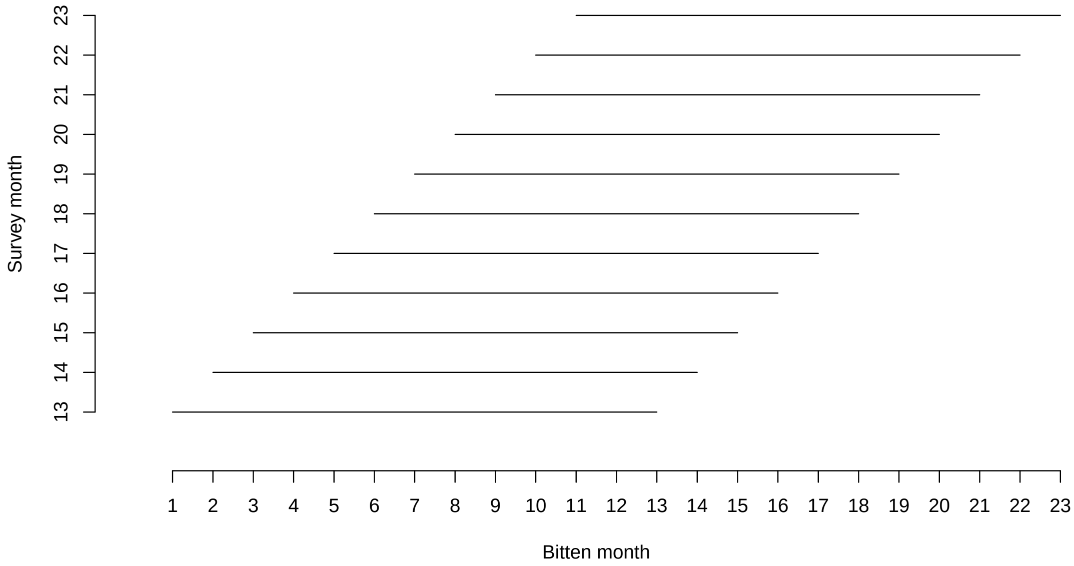

We have previously shown that the pattern of reported snake-bites gives clear evidence of *recall bias*. To account for this, we included withon out model an exponentially decaying function of $s - t$ [1]. Additionally, each $\lambda(s, t)$ must be proportional to the number, $n_{pc}(s)$, of of people sampled in the cluster in question in survey month s. Finally, we assume that each intensity function depend log-linearly on a vector of spatially and/or temporally varying covariates, $x_{pc}(t)$. Putting these elements together gives our model for the intensities $\lambda(s, t)$ as

$$\lambda_{pc}(s,t) = n_{pc}(s) \exp\{-(s-t)\alpha + x_{pc}(t)'\beta\} \tag{3}$$

In equation (3), we included as candidate covariates all of the variables in previously reported separate spatial and temporal models for snake-bite incidence [2, 1]. Spatial covariates at the cluster level included elevation of the cluster centroid, population density, percentage of agricultural workers, average annual rainfall and temperature, modelled as piece-wise linear functions [2]. Temporal covariates included sine and cosine terms with periodicities of 12, 6, 4 and 3 months to represent seasonal trends over the study period, and maximum relative humidity anomaly to account for weather-related departures from the seasonal trend [1]. We fitted separate models to the data for overall and for envenoming snake-bite incidence.

Fitting the model

Parameter estimation and covariate selection used likelihood-based methods as follows.

The generic form for the log-likelihood function of a Poisson process model with intensity $\lambda(t)$

and event-times $t_1, ..., t_n$ observed over a time-interval from 0 to T is [3]

$$L(\lambda) = \sum_{i=1}^{n} \log \lambda(t_i) - \int_0^T \lambda(u)du. \tag{4}$$

We obtain the log-likelihood for each province by adding the separate contributions from all clusters, hence for province p,

$$L_p(\alpha_p, \beta_p) = \sum_{c=1}^{m_p} L(\lambda_{cp}),$$

where the intensities $\lambda_{pc}(t)$ are defined by the combination of equations (1), (2) and (3). The notation indicates that we allow the parameters α and β in (3) to vary between provinces. The log-likelihood for the complete data-set is the sum of the contributions from each province, hence

$$L(\alpha, \beta) = \sum_{p=1}^{9} L_p(\alpha_p, \beta_p),$$

where now $\alpha = (\alpha_1, ..., \alpha_9)$ and $\beta = (\beta_1, ..., \beta_9)$.

We first fitted separate models for each province, then fitted models for the complete data-set under a range of hypotheses about which parameters were common to all nine provinces. In all cases, we estimated the model parameters by maximising the log-likelihood using the Nelder and Mead [4] simplex algorithm as implemented in the `R` function `optim()`.

Exposure variables for each of the provincial models were selected using forward selection based on log-likelihood ratio tests. If L_1 and L_0 denote the maximised values of the log-likelihood for nested models with q_1 and $q_0 < q_1$ parameters, respectively, the log-likelihood ratio statistic is $D = 2(L_1 - L_0)$. If the simpler model is correct, the distribution of D is chi-squared on $q_1 - q_0$ degrees of freedom.

We evaluated the appropriateness of a single recall function for the entire country against models with province-specific recall functions. We adopted province-specific recall functions due to their giving a significant improvement in a generalized likelihood ratio test.

Assessing model fit

Goodness of the fit of the model in each province was evaluated using the following result. If $t_1, ..., t_n$ are the ordered event-times in a Poisson process with intensity $\lambda(t)$ observed over the time-interval from 0 to T then the quantities $v_1, ..., v_n$, where

$$v_i = c^{-1} \int_0^{t_i} \lambda(u)du$$

and $c = \int_0^T \lambda(u)du$, are an ordered random sample from the uniform distribution on the interval 0 to 1. We used the Kolmogorov-Smirnov test to assess the agreement with the uniform distribution.

0.1 Predicting snake-bite incidence

The quantities that we wish to predict are the monthly bite incidences over the whole of Sri Lanka. To do this, note firstly that in equation (3), the modelled risks per person are the quantities

$$\begin{aligned} \rho_{pc}(s,t) &= \lambda(s,t)\exp\{(s-t)\alpha\}/n_{pc}(s) \\ &= n_{pc}(s)\exp\{-(s-t)\alpha + x_{pc}(t)'\beta\} * \exp\{(s-t)\alpha\}/n_{pc}(s,t) \\ &= \exp\{x_{pc}(t)'\beta\} \end{aligned} \quad (5)$$

We represented Sri Lanka as a regular grid of approximately 58,000 points at 0.01 decimal degree resolution and attached to each grid-point the corresponding values of the explanatory variables. We then drew 10,000 predictions of $\rho_{pc}(s,t)$ at each grid-point by sampling from the multivariate Normal distribution of the maximum likelihood parameter estimates in the fitted models. We then prepared probability contour maps (PCMs) to show the spatial variation in the probability that local risk per person does or does not exceed any specified threshold. For the PCMs, we used threshold levels of 39 snakebites per 100,000 people and 19 envenomings per 100,000 people, these being the national average numbers of bites and envenomings, respectively, per 100,000 people per month. Finally, we used our predictions of per-person risk to calculate incidences per 100,000 people for province p in month t as

$$I_p(t) = 100,000 \times \sum_{s=t-12}^{t} \rho_{pc}(s,t), \quad (6)$$

and national incidence per 100,000 people as

$$I(t) = N^{-1} \sum_{p=1}^{9} N_p I_p(t), \quad (7)$$

where N_p is the population of province p and $N = N_1 + N_2 + ... + N_9$. For these calculations, 95% confidence Intervals were again calculated by sampling 10,000 times from the multivariate Normal distribution of the maximum likelihood parameter estimates.

References

[1] D. S. Ediriweera, P. J. Diggle, A. Kasturiratne, A. Pathmeswaran, N. K. Gunawardena, S. F. Jayamanne, G. K. Isbister, A. Dawson, D. G. Lalloo, and H. J. de Silva, "Evaluating temporal patterns of snakebite in sri lanka: the potential for higher snakebite burdens with climate change," *International journal of epidemiology*, vol. 47, no. 6, pp. 2049–2058, 2018.

[2] D. S. Ediriweera, A. Kasturiratne, A. Pathmeswaran, N. K. Gunawardena, B. A. Wijayawickrama, S. F. Jayamanne, G. K. Isbister, A. Dawson, E. Giorgi, P. J. Diggle, *et al.*,

"Mapping the risk of snakebite in sri lanka-a national survey with geospatial analysis," *PLoS neglected tropical diseases*, vol. 10, no. 7, p. e0004813, 2016.

[3] P. J. Diggle, *Statistical analysis of spatial and spatio-temporal point patterns.* Chapman and Hall/CRC, 2013.

[4] J. A. Nelder and R. Mead, "A simplex method for function minimization," *The computer journal*, vol. 7, no. 4, pp. 308–313, 1965.
