# Supplementary material for: Evaluating spatiotemporal dynamics of snakebite in Sri Lanka: Monthly incidence mapping from a national representative survey sample
Source: PLoS Negl Trop Dis. 2021 Jun 1;15(6):e0009447. doi: 10.1371/journal.pntd.0009447 (PMC8195360; doi:10.1371/journal.pntd.0009447)
Supplement: S5 Appendix — (DOCX) [file pntd.0009447.s005.docx]

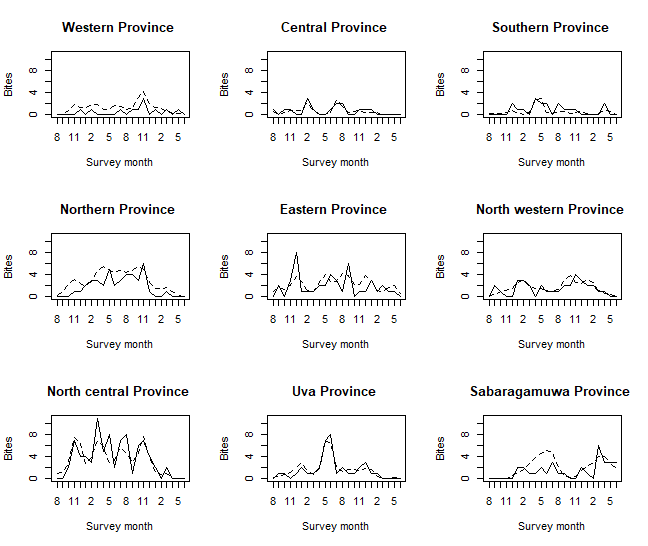


**S5 Appendix: Observed snakebites versus predicted snakebites at each province.**

Solid lines indicate observed snakebites and dashed lines indicate predicted snakebites for the survey sample at each province.
