# Supplementary material for: Evaluating spatiotemporal dynamics of snakebite in Sri Lanka: Monthly incidence mapping from a national representative survey sample
Source: PLoS Negl Trop Dis. 2021 Jun 1;15(6):e0009447. doi: 10.1371/journal.pntd.0009447 (PMC8195360; doi:10.1371/journal.pntd.0009447)
Supplement: S6 Appendix — (DOCX) [file pntd.0009447.s006.docx]

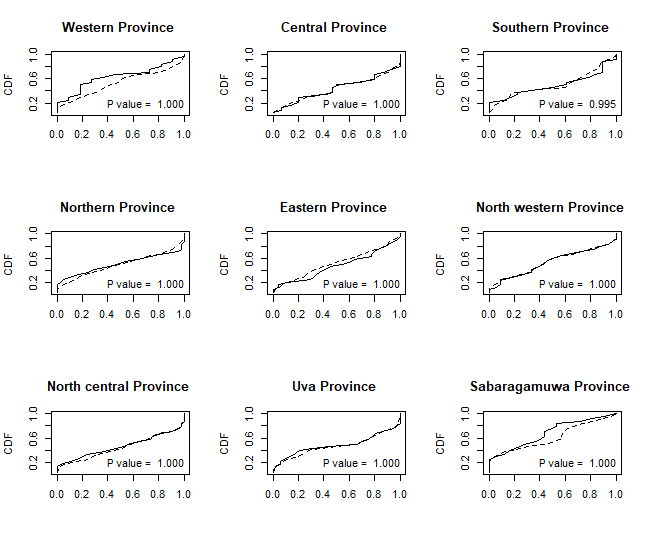


**S6 Appendix. Goodness of fit tests.** Observed (solid line) and predicted (dashed line) envenoming bites for the survey sample with probability integral transformation. P values are from Kolmogorov-Smirnov tests.
