# Supplementary material for: Evaluating spatiotemporal dynamics of snakebite in Sri Lanka: Monthly incidence mapping from a national representative survey sample
Source: PLoS Negl Trop Dis. 2021 Jun 1;15(6):e0009447. doi: 10.1371/journal.pntd.0009447 (PMC8195360; doi:10.1371/journal.pntd.0009447)
Supplement: S1 Table — (DOCX) [file pntd.0009447.s007.docx]

**S1 Table: Parameter estimates and standard errors at each province level for snakebites**

| Western Province | Parameter estimate | Standard Error |
| --- | --- | --- |
| Intercept | - 1.65 | 80.46 |
| Sin(12t) | + 0.22 | 0.21 |
| Cosine(12t) | - 0.13 | 0.18 |
| Sin(6t) | - 0.11 | 0.18 |
| Cosine(6t) | - 0.20 | 0.20 |
| Sin(4t) | + 0.01 | 0.19 |
| Cosine(4t) | + 0.16 | 0.19 |
| Sin(3t) | + 0.37 | 0.19 |
| Cosine(3t) | - 0.21 | 0.19 |
| Rainfall | - 0.44 | 1.93 |
| Temperature | - 0.59 | 20.87 |
| Population density | - 0.36 | 0.19 |
| Agriculture | + 0.31 | 0.18 |
| Agriculture > 9% | + 0.02 | 0.07 |
| Recall | - 0.01 | 0.03 |

| Central Province | Parameter estimate | Standard Error |
| --- | --- | --- |
| Intercept | - 0.41 | 9.75 |
| Sin(12t) | + 0.29 | 0.27 |
| Cosine(12t) | - 0.12 | 0.20 |
| Sin(6t) | - 0.03 | 0.21 |
| Cosine(6t) | - 0.55 | 0.26 |
| Sin(4t) | + 0.02 | 0.23 |
| Cosine(4t) | + 0.08 | 0.22 |
| Sin(3t) | + 0.22 | 0.22 |
| Cosine(3t) | - 0.33 | 0.22 |
| Rainfall | - 1.08 | 1.28 |
| Population density | - 0.39 | 0.11 |
| Recall | - 0.05 | 0.04 |

| Southern Province | Parameter estimate | Standard Error |
| --- | --- | --- |
| Intercept | - 9.18 | 3.70 |
| Sin(12t) | + 0.06 | 0.14 |
| Cosine(12t) | - 0.26 | 0.18 |
| Sin(6t) | - 0.01 | 0.15 |
| Cosine(6t) | - 0.30 | 0.17 |
| Sin(4t) | + 0.35 | 0.15 |
| Cosine(4t) | - 0.26 | 0.16 |
| Sin(3t) | - 0.12 | 0.15 |
| Cosine(3t) | + 0.21 | 0.15 |
| Rainfall | - 0.37 | 0.10 |
| Elevation | + 0.38 | 0.49 |
| Recall | + 0.01 | 0.03 |

| Northern Province | Parameter estimate | Standard Error |
| --- | --- | --- |
| Intercept | - 10.30 | 0.33 |
| Sin(12t) | + 0.19 | 0.19 |
| Cosine(12t) | + 0.07 | 0.20 |
| Sin(6t) | - 0.28 | 0.18 |
| Cosine(6t) | - 0.21 | 0.21 |
| Sin(4t) | + 0.08 | 0.19 |
| Cosine(4t) | + 0.08 | 0.19 |
| Sin(3t) | + 0.03 | 0.19 |
| Cosine(3t) | - 0.36 | 0.19 |
| Agriculture | + 0.12 | 0.18 |
| Agriculture >9% | + 0.02 | 0.08 |
| Recall | - 0.06 | 0.03 |

| Eastern Province | Parameter estimate | Standard Error |
| --- | --- | --- |
| Intercept | - 1.45 | 22.26 |
| Sin(12t) | - 0.02 | 0.19 |
| Cosine(12t) | - 0.33 | 0.20 |
| Sin(6t) | - 0.12 | 0.18 |
| Cosine(6t) | - 0.13 | 0.20 |
| Sin(4t) | + 0.25 | 0.18 |
| Cosine(4t) | - 0.24 | 0.17 |
| Sin(3t) | -0.28 | 0.18 |
| Cosine(3t) | - 0.01 | 0.17 |
| Rainfall | - 0.91 | 2.99 |
| Population density | - 0.24 | 0.07 |
| Agriculture >9% | + 0.64 | 0.19 |
| Recall | - 0.01 | 0.04 |

| North Western Province | Parameter estimate | Standard Error |
| --- | --- | --- |
| Intercept | - 3.69 | 9.73 |
| Sin(12t) | + 0.01 | 0.15 |
| Cosine(12t) | - 0.21 | 0.16 |
| Sin(6t) | - 0.02 | 0.14 |
| Cosine(6t) | - 0.14 | 0.16 |
| Sin(4t) | - 0.07 | 0.15 |
| Cosine(4t) | - 0.22 | 0.15 |
| Sin(3t) | + 0.18 | 0.15 |
| Cosine(3t) | - 0.28 | 0.15 |
| Elevation | + 0.67 | 0.17 |
| Rainfall | - 1.37 | 1.40 |
| Agriculture | - 0.41 | 0.31 |
| Agriculture >9% | - 0.02 | 0.06 |
| Recall | - 0.05 | 0.03 |

| North Central Province | Parameter estimate | Standard Error |
| --- | --- | --- |
| Intercept | + 0.33 | 10.12 |
| Sin(12t) | + 0.02 | 0.14 |
| Cosine(12t) | - 0.19 | 0.15 |
| Sin(6t) | - 0.15 | 0.13 |
| Cosine(6t) | - 0.21 | 0.13 |
| Sin(4t) | + 0.29 | 0.13 |
| Cosine(4t) | + 0.37 | 0.13 |
| Sin(3t) | + 0.29 | 0.13 |
| Cosine(3t) | - 0.01 | 0.13 |
| Rainfall | - 1.37 | 1.38 |
| Agriculture >9% | + 0.51 | 0.17 |
| Recall | + 0.01 | 0.02 |

| Uva Province | Parameter estimate | Standard Error |
| --- | --- | --- |
| Intercept | - 0.39 | 13.11 |
| Sin(12t) | – 0.11 | 0.21 |
| Cosine(12t) | - 0.01 | 0.19 |
| Sin(6t) | - 0.35 | 0.18 |
| Cosine(6t) | + 0.14 | 0.20 |
| Sin(4t) | + 0.02 | 0.19 |
| Cosine(4t) | - 0.34 | 0.19 |
| Sin(3t) | - 0.06 | 0.19 |
| Cosine(3t) | - 0.26 | 0.19 |
| Rainfall | - 1.16* | 0.02 |
| Elevation >195m | - 0.01 | 1.76 |
| Population density | - 0.35 | 0.11 |
| Recall | - 0.01 | 0.03 |

| Sabaragamuwa Province | Parameter estimate | Standard Error |
| --- | --- | --- |
| Intercept | - 3.37 | 4.65 |
| Sin(12t) | – 0.20 | 0.16 |
| Cosine(12t) | + 0.04 | 0.15 |
| Sin(6t) | - 0.13 | 0.13 |
| Cosine(6t) | - 0.05 | 0.17 |
| Sin(4t) | + 0.49 | 0.15 |
| Cosine(4t) | + 0.02 | 0.15 |
| Sin(3t) | + 0.10 | 0.15 |
| Cosine(3t) | -0.37 | 0.15 |
| Rainfall | - 0.70 | 0.60 |
| Population density | - 0.23 | 0.12 |
| Recall | - 0.04 | 0.03 |
