# Supplementary material for: Evaluating spatiotemporal dynamics of snakebite in Sri Lanka: Monthly incidence mapping from a national representative survey sample
Source: PLoS Negl Trop Dis. 2021 Jun 1;15(6):e0009447. doi: 10.1371/journal.pntd.0009447 (PMC8195360; doi:10.1371/journal.pntd.0009447)
Supplement: S2 Table — (DOCX) [file pntd.0009447.s008.docx]

**S2 Table: Parameter estimates and standard errors at each province level for envenoming bites**

| Western Province | Parameter estimate | Standard Error |
| --- | --- | --- |
| Intercept | - 1.36 | 103.51 |
| Sin(12t) | + 0.27 | 0.50 |
| Cosine(12t) | - 0.51 | 0.50 |
| Sin(6t) | - 0.21 | 0.49 |
| Cosine(6t) | - 0.05 | 0.49 |
| Sin(4t) | + 0.27 | 0.46 |
| Cosine(4t) | + 0.34 | 0.47 |
| Temperature | - 2.41 | 31.62 |
| Population density | - 0.33 | 0.35 |
| Recall | - 0.19 | 0.01 |

| Central Province | Parameter estimate | Standard Error |
| --- | --- | --- |
| Intercept | + 0.13 | 16.44 |
| Sin(12t) | + 0.93 | 0.70 |
| Cosine(12t) | - 0.34 | 0.43 |
| Sin(6t) | - 0.66 | 0.49 |
| Cosine(6t) | - 0.97 | 0.52 |
| Sin(4t) | - 0.30 | 0.44 |
| Cosine(4t) | + 0.85 | 0.48 |
| Rainfall | -1.21 | 0.19 |
| Population density | - 0.66 | 2.18 |
| Recall | + 0.03 | 0.08 |

| Southern Province | Parameter estimate | Standard Error |
| --- | --- | --- |
| Intercept | - 3.17 | 10.51 |
| Sin(12t) | - 0.22 | 0.36 |
| Cosine(12t) | + 0.07 | 0.39 |
| Sin(6t) | - 0.28 | 0.36 |
| Cosine(6t) | - 0.38 | 0.37 |
| Sin(4t) | + 0.59 | 0.37 |
| Cosine(4t) | - 0.29 | 0.36 |
| Rainfall | - 2.03 | 0.08 |
| Elevation >195m | + 0.13 | 0.10 |
| Agriculture >9% | + 0.04 | 1.40 |
| Recall | + 0.12 | 0.07 |

| Northern Province | Parameter estimate | Standard Error |
| --- | --- | --- |
| Intercept | + 0.67 | 33.08 |
| Sin(12t) | + 0.07 | 0.22 |
| Cosine(12t) | + 0.05 | 0.24 |
| Sin(6t) | - 0.07 | 0.23 |
| Cosine(6t) | - 0.23 | 0.23 |
| Sin(4t) | + 0.02 | 0.22 |
| Cosine(4t) | + 0.16 | 0.22 |
| Rainfall | - 1.51 | 0.15 |
| Agriculture | + 0.29 | 4.57 |
| Recall | - 0.06 | 0.05 |

| Eastern Province | Parameter estimate | Standard Error |
| --- | --- | --- |
| Intercept | - 4.13 | 27.01 |
| Sin(12t) | + 0.21 | 0.24 |
| Cosine(12t) | - 0.12 | 0.22 |
| Sin(6t) | - 0.23 | 0.23 |
| Cosine(6t) | + 0.11 | 0.22 |
| Sin(4t) | + 0.30 | 0.22 |
| Cosine(4t) | - 0.24 | 0.22 |
| Rainfall | - 0.51 | 0.10 |
| Population density | - 0.30 | 3.87 |
| Agriculture | - 2.35 | 2.76 |
| Agriculture > 9% | + 2.64 | 3.63 |
| Recall | - 0.01 | 0.05 |

| North Western Province | Parameter estimate | Standard Error |
| --- | --- | --- |
| Intercept | -5.09 | 13.06 |
| Sin(12t) | + 0.26 | 0.27 |
| Cosine(12t) | - 0.66 | 0.30 |
| Sin(6t) | + 0.16 | 0.28 |
| Cosine(6t) | - 0.09 | 0.29 |
| Sin(4t) | - 0.19 | 0.26 |
| Cosine(4t) | - 0.19 | 0.26 |
| Rainfall | - 1.02 | 0.18 |
| Population density | + 0.16 | 1.82 |
| Recall | + 0.01 | 0.05 |

| North Central Province | Parameter estimate | Standard Error |
| --- | --- | --- |
| Intercept | -1.92 | 13.52 |
| Sin(12t) | + 0.13 | 0.17 |
| Cosine(12t) | - 0.13 | 0.17 |
| Sin(6t) | - 0.11 | 0.165 |
| Cosine(6t) | - 0.15 | 0.16 |
| Sin(4t) | + 0.19 | 0.16 |
| Cosine(4t) | + 0.44 | 0.16 |
| Rainfall | -1.32 | 0.33 |
| Elevation | + 0.36 | 0.04 |
| Elevation >195m | + 0.08 | 0.21 |
| Agriculture >9% | + 0.41 | 1.78 |
| Recall | + 0.05 | 0.03 |

| Uva Province | Parameter estimate | Standard Error |
| --- | --- | --- |
| Intercept | -0.14 | 18.19 |
| Sin(12t) | – 0.12 | 0.29 |
| Cosine(12t) | + 0.16 | 0.26 |
| Sin(6t) | - 0.65 | 0.27 |
| Cosine(6t) | + 0.14 | 0.25 |
| Sin(4t) | - 0.16 | 0.26 |
| Cosine(4t) | - 0.42 | 0.26 |
| Rainfall | -1.49 | 2.45 |
| Agriculture | + 0.42 | 0.33 |
| Recall | + 0.01 | 0.05 |

| Sabaragamuwa Province | Parameter estimate | Standard Error |
| --- | --- | --- |
| Intercept | -5.67 | 8.61 |
| Sin(12t) | - 1.36 | 0.50 |
| Cosine(12t) | + 0.06 | 0.30 |
| Sin(6t) | - 0.02 | 0.34 |
| Cosine(6t) | + 0.56 | 0.41 |
| Sin(4t) | + 0.26 | 0.31 |
| Cosine(4t) | + 0.08 | 0.30 |
| Rainfall | - 0.56* | 0.26 |
| Agriculture | + 0.72* | 1.15 |
| Recall | - 0.05* | 0.05 |
