# Supplementary material for: Evaluating spatiotemporal dynamics of snakebite in Sri Lanka: Monthly incidence mapping from a national representative survey sample
Source: PLoS Negl Trop Dis. 2021 Jun 1;15(6):e0009447. doi: 10.1371/journal.pntd.0009447 (PMC8195360; doi:10.1371/journal.pntd.0009447)
Supplement: Attachment — Submitted filename: Response to reviewers.docx [file pntd.0009447.s011.docx]

Associate Editor and Deputy Editor

PLOS Neglected Tropical Diseases

15/01/2021

Dear Madam/Sir,

**RE: Resubmission of revised manuscript**

Thank you very much for reviewing our manuscript "Evaluating spatiotemporal dynamics of snakebite in Sri Lanka: monthly incidence mapping from a national representative survey sample" for consideration at PLOS Neglected Tropical Diseases.

We have now responded the reviewer comments and update our manuscript accordingly. Herewith I am submitting a letter with the responses to review comments and two version of revised manuscripts (i.e. a clean copy and a copy with tracked changes).

In addition, we noticed that there were intext comments by the reviewers and we have responded to those comments within the text (which can be found at the “Manuscript with tracked changes”).

Thank you

Sincerely

Dileepa Ediriweera

Dear Dr Ediriweera,,
 
Thank you very much for submitting your manuscript "Evaluating spatiotemporal dynamics of snakebite in Sri Lanka: monthly incidence mapping from a national representative survey sample" for consideration at PLOS Neglected Tropical Diseases. As with all papers reviewed by the journal, your manuscript was reviewed by members of the editorial board and by several independent reviewers. In light of the reviews (below this email), we would like to invite the resubmission of a significantly-revised version that takes into account the reviewers' comments.
 

We cannot make any decision about publication until we have seen the revised manuscript and your response to the reviewers' comments. Your revised manuscript is also likely to be sent to reviewers for further evaluation.
 
When you are ready to resubmit, please upload the following:

[1] A letter containing a detailed list of your responses to the review comments and a description of the changes you have made in the manuscript. Please note while forming your response, if your article is accepted, you may have the opportunity to make the peer review history publicly available. The record will include editor decision letters (with reviews) and your responses to reviewer comments. If eligible, we will contact you to opt in or out.

[2] Two versions of the revised manuscript: one with either highlights or tracked changes denoting where the text has been changed; the other a clean version (uploaded as the manuscript file).

Important additional instructions are given below your reviewer comments.

Please prepare and submit your revised manuscript within 60 days. If you anticipate any delay, please let us know the expected resubmission date by replying to this email. Please note that revised manuscripts received after the 60-day due date may require evaluation and peer review similar to newly submitted manuscripts.
 
Thank you again for your submission. We hope that our editorial process has been constructive so far, and we welcome your feedback at any time. Please don't hesitate to contact us if you have any questions or comments.
 
Sincerely,

Marilia Sá Carvalho
Associate Editor
PLOS Neglected Tropical Diseases

José Gutiérrez
Deputy Editor
PLOS Neglected Tropical Diseases

***********************


Reviewer's Responses to Questions

<b>Key Review Criteria Required for Acceptance?</b></br></br>
As you describe the new analyses required for acceptance, please consider the following:</br></br>

<b>Methods</b></br></br>
-Are the objectives of the study clearly articulated with a clear testable hypothesis stated?</br>
-Is the study design appropriate to address the stated objectives?</br>
-Is the population clearly described and appropriate for the hypothesis being tested?</br>
-Is the sample size sufficient to ensure adequate power to address the hypothesis being tested?</br>
-Were correct statistical analysis used to support conclusions?</br>
-Are there concerns about ethical or regulatory requirements being met?</br></br>

Reviewer #1: The methodology used was consistent with the results achieved.
It's all ok!

- Thank you.

Reviewer #2: -Are the objectives of the study clearly articulated with a clear testable hypothesis stated? Need to improve.

- We have now updated the text (lines 114 – 121) and responded to the relevant comments in the manuscript.

-Is the study design appropriate to address the stated objectives? It has some critical points, but it is acceptable considering the place of study.
-Is the population clearly described and appropriate for the hypothesis being tested? I suggest that he explain better the criteria for choosing the interviewed population.

- We have now updated the text (lines 141 - 178) and responded to the relevant comments in the manuscript.

-Is the sample size sufficient to ensure adequate power to address the hypothesis being tested? Yes.
-Were correct statistical analysis used to support conclusions? Yes.
-Are there concerns about ethical or regulatory requirements being met? No.

- Thank you.

Reviewer #3: The study shows objectives articulated with a hypothesis
The study design is appropriate
The population is clearly described
The sample size is god
There is a correct statistical analysis
The study was approved by ethical committees

- Thank you.

--------------------

<b>Results</b></br></br>
-Does the analysis presented match the analysis plan?</br>
-Are the results clearly and completely presented?</br>
-Are the figures (Tables, Images) of sufficient quality for clarity?</br></br>

Reviewer #1: It's all ok!

- Thank you.

Reviewer #2: -Does the analysis presented match the analysis plan? Yes.
-Are the results clearly and completely presented? It can improve a lot.

- We have now updated the text (lines 243 - 327) and responded to the relevant comments in the manuscript.

-Are the figures (Tables, Images) of sufficient quality for clarity? I found the tables a little confusing, but the images are ok.

- We accept that these tables are complex and do not follow the conventional table formats. Therefore, we only presented the summary of the tables in the manuscript and presented the details in the supplementary materials to avoid further complexity.
- I am afraid to mention that, we are unable to further simplify these tables.

Reviewer #3: The analysis presented does match the analysis plan; The results are clearly and completely presented and figures (Tables, Images) are relevant, with quality and clarity

- Thank you.

--------------------

<b>Conclusions</b></br></br>
-Are the conclusions supported by the data presented?</br>
-Are the limitations of analysis clearly described?</br>
-Do the authors discuss how these data can be helpful to advance our understanding of the topic under study?</br>
-Is public health relevance addressed?</br></br>

Reviewer #1: It's all ok!

- Thank you.

Reviewer #2: -Are the conclusions supported by the data presented? Yes.
-Are the limitations of analysis clearly described? Yes.
-Do the authors discuss how these data can be helpful to advance our understanding of the topic under study? Yes.
-Is public health relevance addressed? Yes.

- Thank you.

Reviewer #3: From the perspective of my role in the confluence between animals of medical and bioethical interest, I believe that there could be an innovation in the approach of the issue. I'll point out a few points.

- First, the authors consider it to be auxiliary in the determination of hotspots to target the service. I believe these strategies could be discussed with the community. In the matter of health control and animal accidents the community needs to see as a problem to help in the resolution. Thus, the existence of committees with popular participation, the academy and the management bodies could take advantage of the data in a more applied and real way. In this context I suggest the insertion of paradigms and perspectives of bioethics. Bioethics identifies vulnerabilities, listens to arguments and proposes solutions taken together and that are consensual and fair for all.
  - Thank you for the suggestion, we have included this aspect in the discussion (lines 336 - 339)
- the second point I highlight tourism, which in my perspective is an important point for the country. therefore, the knowledge of hotspots could be determinant in management plans for sustainable use of natural areas. consequently, the accidents themselves, the relationship of animal residents and the reduction of accidents could be achieved with environmental education processes. I point out that the work as it is brings an important technical contribution with data relevant to decision making, which in itself are sufficient. However, this suggestion is optional and I believe it could help bring a new perspective to the area.
  - Thank you for the suggestion, we have included this aspect in the discussion (lines 388 - 391)

--------------------

<b>Editorial and Data Presentation Modifications?</b></br><br/>
Use  this section for editorial suggestions as well as relatively minor modifications of existing data that would enhance clarity. If the only modifications needed are minor and/or editorial, you may wish to recommend “Minor Revision” or “Accept”.

Reviewer #1: Insert the following reference in the correct way below:
França FGR, Braz VS.. Diversity, activity patterns, and habitat use of the snake fauna of Chapada dos Veadeiros National Park in Central Brazil. Biota Neotrop. 2013; 13:74–85. doi:10.1590/S1676-06032013000100008

- Thank you fro pointing out the issue and we have updated the reference now.

Reviewer #2: I believe that the larger images would be more readable.

- Thank you for the suggestion, we have now provided the figures separately in accordance with the Preflight Analysis and Conversion Engine (PACE) requirements.

Reviewer #3: From the perspective of my role in the confluence between animals of medical and bioethical interest, I believe that there could be an innovation in the approach of the issue. I'll point out a few points. First, the authors consider it to be auxiliary in the determination of hotspots to target the service. I believe these strategies could be discussed with the community. In the matter of health control and animal accidents the community needs to see as a problem to help in the resolution. Thus, the existence of committees with popular participation, the academy and the management bodies could take advantage of the data in a more applied and real way. In this context I suggest the insertion of paradigms and perspectives of bioethics. Bioethics identifies vulnerabilities, listens to arguments and proposes solutions taken together and that are consensual and fair for all.

- Thank you for the suggestion, we have included this aspect in the discussion (lines 336 - 339)

the second point I highlight tourism, which in my perspective is an important point for the
country. therefore, the knowledge of hotspots could be determinant in management plans for sustainable use of natural areas. consequently, the accidents themselves, the relationship of animal residents and the reduction of accidents could be achieved with environmental education processes. I point out that the work as it is brings an important technical contribution with data relevant to decision making, which in itself are sufficient. However, this suggestion is optional and I believe it could help bring a new perspective to the area.

- Thank you for the suggestion, we have included this aspect in the discussion (lines lines 388 - 391)

--------------------

<b>Summary and General Comments</b></br></br>
Use  this section to provide overall comments, discuss strengths/weaknesses of the study, novelty, significance, general execution and scholarship. You may also include additional comments for the author, including concerns about dual publication, research ethics, or publication ethics. If requesting major revision, please articulate the new experiments that are needed.

Reviewer #1: It's all ok!

Reviewer #2: Most of my considerations are in the text. I would just like to add, that if possible, make the tables easier to understand.

- We have responded to the relevant comments in the manuscript (please refer the manuscript with tracked changes).
- We accept that these tables are complex and not follow the conventional tables. Therefore, we only presented the summary of the tables in the manuscript and presented the details in the supplementary materials to avoid further complexity.
- I am afraid to mention that, we are unable to further simplify these tables.

Reviewer #3: From the perspective of my role in the confluence between animals of medical and bioethical interest, I believe that there could be an innovation in the approach of the issue. I'll point out a few points. First, the authors consider it to be auxiliary in the determination of hotspots to target the service. I believe these strategies could be discussed with the community. In the matter of health control and animal accidents the community needs to see as a problem to help in the resolution. Thus, the existence of committees with popular participation, the academy and the management bodies could take advantage of the data in a more applied and real way. In this context I suggest the insertion of paradigms and perspectives of bioethics. Bioethics identifies vulnerabilities, listens to arguments and proposes solutions taken together and that are consensual and fair for all.

- Thank you for the suggestion, we have included this aspect in the discussion (lines 336 - 339)

the second point I highlight tourism, which in my perspective is an important point for the
country. therefore, the knowledge of hotspots could be determinant in management plans for sustainable use of natural areas. consequently, the accidents themselves, the relationship of animal residents and the reduction of accidents could be achieved with environmental education processes. I point out that the work as it is brings an important technical contribution with data relevant to decision making, which in itself are sufficient. However, this suggestion is optional and I believe it could help bring a new perspective to the area.

- Thank you for the suggestion, we have included this aspect in the discussion (lines lines 388 - 391)

--------------------

PLOS authors have the option to publish the peer review history of their article (<a href="<https://journals.plos.org/plosntds/s/editorial-and-peer-review-process#loc-peer-review-history>" target="_blank">what does this mean?</a>). If published, this will include your full peer review and any attached files.<br><br>

If you choose “no”, your identity will remain anonymous but your review may still be made public.<br><br>

<b>Do you want your identity to be public for this peer review?</b> For information about this choice, including consent withdrawal, please see our <a href="<https://www.plos.org/privacy-policy>" target="_blank">Privacy Policy</a>.

Reviewer #1: Yes: Paulo Sérgio Bernarde

Reviewer #2: Yes: Gabriela Ferreira Campos Guerra

Reviewer #3: Yes: Marta Luciane Fischer


Figure Files:
While revising your submission, please upload your figure files to the Preflight Analysis and Conversion Engine (PACE) digital diagnostic tool, [https://pacev2.apexcovantage.com](https://pacev2.apexcovantage.com/). PACE helps ensure that figures meet PLOS requirements. To use PACE, you must first register as a user. Then, login and navigate to the UPLOAD tab, where you will find detailed instructions on how to use the tool. If you encounter any issues or have any questions when using PACE, please email us at [figures@plos.org](mailto:figures@plos.org).
 
Data Requirements:
Please note that, as a condition of publication, PLOS' data policy requires that you make available all data used to draw the conclusions outlined in your manuscript. Data must be deposited in an appropriate repository, included within the body of the manuscript, or uploaded as supporting information. This includes all numerical values that were used to generate graphs, histograms etc.. For an example see here: <http://www.plosbiology.org/article/info%3Adoi%2F10.1371%2Fjournal.pbio.1001908#s5>.
 
Reproducibility:
To enhance the reproducibility of your results, PLOS recommends that you deposit laboratory protocols in [protocols.io](http://protocols.io/), where a protocol can be assigned its own identifier (DOI) such that it can be cited independently in the future. For instructions see <https://journals.plos.org/plosntds/s/submission-guidelines#loc-methods>
